# Caregiving-Related Depression and Neuroinflammation: A Longitudinal Study

**DOI:** 10.1093/geroni/igaf122.1352

**Published:** 2025-12-31

**Authors:** So Yeon Jeon, Bo Ran Son, Hee Won Yang, Jimin Baek, Jeong Lan Kim

**Affiliations:** Seoul Metropolitan Government-Seoul National University (SMG-SNU) Boramae Medical Center, Seoul, Republic of Korea; Chungnam National University Hospital, Daejeon, Republic of Korea; Chungnam National University Hospital, Daejeon, Republic of Korea; Chungnam National University Hospital, Daejeon, Republic of Korea; Chungnam National University Hospital, Daejeon, Republic of Korea

## Abstract

**Background:**

The caregiving burden of the spousal caregivers (SCGs) to individuals with cognitive impairment poses public health challenges with adverse psychosocial and physiological effects. However, few studies have investigated the neurobiological impact of caregiving, particularly through the investigation of neuroinflammation and neurodegeneration.

**Methods:**

Using data from a longitudinal cohort at Chungnam National University Hospital, the relationship between caregiving burden, neuroinflammation and neurodegeneration was examined in 38 older adult couples over a 16-month period. Caregiving burden was assessed through a multifaceted approach. For factors related to the care recipient, we assessed cognitive function and neuropsychiatric symptoms. Factors regarding the SCGs included the measurement of perceived depression. Glial fibrillary acidic protein (GFAP) was used as a plasma biomarker for neuroinflammation and neurofilament light chain (NfL) for neurodegeneration. Regression analyses were adjusted for age, sex, apolipoprotein E status, follow-up interval, vascular risk factors, and physical activity.

**Results:**

Changes in depression among SCGs were significantly correlated with increased GFAP levels (p = 0.003), indicating that greater depressive symptoms during caregiving are associated with increased neuroinflammation. In contrast, no significant correlations were found between changes in cognitive function or neuropsychiatric symptoms in care recipients and the plasma biomarker levels of SCGs. Additionally, there was no significant association between changes in depression and NfL levels in SCGs.

**Conclusions:**

The psychological stress experienced by SCGs while caring for partners with cognitive impairment actively contributes to neuroinflammation, a well-known risk factor for various diseases. This study emphasizes the need to address psychological stress experienced by older adult caregivers.

